# Prevalence and determinants of meeting minimum dietary diversity among children aged 6–23 months in three sub-Saharan African Countries: The Demographic and Health Surveys, 2019–2020

**DOI:** 10.3389/fpubh.2022.846049

**Published:** 2022-08-23

**Authors:** Djibril M. Ba, Paddy Ssentongo, Xiang Gao, Vernon M. Chinchilli, John P. Richie, Mamoudou Maiga, Joshua E. Muscat

**Affiliations:** ^1^Department of Public Health Sciences, Penn State College of Medicine, Hershey, PA, United States; ^2^Department of Nutritional Sciences, Penn State University, State College, PA, United States; ^3^Department of Nutrition and Food Hygiene, School of Public Health, Fudan University, Shanghai, China; ^4^Northwestern University, Department of Biomedical Engineering, Evanston, IL, United States

**Keywords:** sub-Saharan Africa, dietary diversity, children, nutrition, DHS

## Abstract

**Background:**

Dietary diversity is an indicator of nutritional adequacy, which plays a significant role in child growth and development. Lack of adequate nutrition is associated with suboptimal brain development, lower school performance, and increased risk of mortality and chronic diseases. We aimed to determine the prevalence and determinants of meeting minimum dietary diversity (MDD), defined as consuming at least five out of eight basic food groups in the previous 24-h in three sub-Saharan African countries.

**Methods:**

A weighted population-based cross-sectional study was conducted using the most recent Demographic and Health Surveys (DHS). MDD data were available between 2019 and 2020 for three sub-Saharan African countries (Gambia, Liberia, and Rwanda). The study population included 5,832 children aged 6–23 months. A multivariable logistic regression model was developed to identify independent factors associated with meeting MDD.

**Results:**

Overall, the weighted prevalence of children who met the MDD was 23.2% (95% CI: 21.7–24.8%), ranging from 8.6% in Liberia to 34.4% in Rwanda. Independent factors associated with meeting MDD were: age of the child (OR) = 1.96, 95% CI: 1.61, 2.39 for 12–17 months vs. 6–11 months], mothers from highest households' wealth status (OR = 1.86, 95% CI: 1.45–2.39) compared with the lowest, and mothers with secondary/higher education (OR = 1.69, 95% CI: 1.35–2.12) compared with those with no education. Mothers who were employed, had access to a radio, and those who visited a healthcare facility in the last 12 months were more likely to meet the MDD. There was no significant association between the child's sex and the odds of fulfilling the MDD.

**Conclusions:**

There is substantial heterogeneity in the prevalence of MDD in these three sub-Saharan African countries. Lack of food availability or affordability may play a significant role in the low prevalence of MDD. The present analysis suggests that policies that will effectively increase the prevalence of meeting MDD should target poor households with appropriate materials or financial assistance and mothers with lower literacy. Public health interventions working with sectors such as education and radio stations to promote health education about the benefits of diverse diets is a critical step toward improving MDD in sub-Saharan Africa and preventing undernutrition.

## Introduction

Undernutrition has decreased globally but remains endemic in several regions such as southeastern Asia and sub-Saharan Africa (SSA) (1, 2). Between 2000 and 2020, the number of children affected by stunting under age 5 worldwide declined from 203.6 million to 149.2 million (3). However, during the same time, the numbers have increased at an alarming rate in SSA—from 22.8 million to 29.3 million (3). Child undernutrition is an important cause of preventable disease burden of public health significance affecting children, specifically those living in SSA (4). Lack of adequate nutrition is associated with inadequate brain development, lower school performance, increased risk of mortality, and chronic diseases (5). According to the United Nations Children's Fund (UNICEF), about 3.1 million children die from undernutrition each year (6). Globally, in 2020, approximately 45.4 million children under age 5 were wasted and 38.9 million were overweight (7). According to the WHO, the risk of a child dying before reaching 5 years of age in Africa is nearly 8 times higher than in Europe (76.5 per 1,000 live births vs. 9.6 per 1,000 live births) (8). Child dietary diversity has been shown to be positively associated with the mean micronutrient adequacy of the diet (9, 10). Therefore, the minimum dietary diversity (MDD) can be effective in assessing a population-level picture of infant and young child diet quality and appropriate complementary feeding practices in low resource settings such as SSA. Enhanced child feeding practices by providing adequately diversified food such as meeting MDD can result in improved energy and nutrient intake, which can lead to better nutritional status and children's overall health and well-being (11).

The MDD score is a population-level indicator developed by the World Health Organization (WHO) to assess diet diversity as part of infant and young child feeding (IYCF) practices among children 6–23 months old (12). The WHO has recommended that a child consumes the MDD of ≥ 5 of 8 pre-defined food groups during the previous 24-h to meet daily energy and essential nutrients requirements (13). Diversified diet assists children to have the proper nutrients needed to maintain optimal child growth and development during critical periods (14, 15). A diverse diet is more likely to meet both macro-and micronutrient needs for human health (16).

According to previous studies, sociodemographic-economic factors such as household wealth index (17–20), maternal age (20), maternal education (18, 20, 21) maternal employment status (20, 22, 23), contact with health care facility (21), place of residence (22, 24), and exposure to mass media such as radio (17, 18, 20) have been suggested to affect MDD among children aged 6–23 months in low-and middle-income countries (LMICs) including SSA. In addition, child factor such as age has also been associated with MDD in SSA (19, 20, 24). To improve the proportion of children fed with a diet meeting MDD in SSA, it is essential to fully understand regional and country-specific variations in the prevalence of MDD and associated factors. Such knowledge will assist in putting in place regional initiatives to prioritize intervention strategies for the most at-risk countries in SSA and assist stakeholders to adequately identifying potential contributing factors for the low prevalence of meeting MDD. However, these estimates are lacking because most previous studies that have examined the determinants of meeting MDD in SSA such as access to media, level of education, and wealth status were mainly limited to individual countries such as Ethiopia (17, 25, 26). Thus, we aimed to fill this critical gap in our knowledge by conducting a multi-country population-based cross-sectional study of the prevalence of meeting MDD in three combined SSA countries; and examining the associated socio-demographic-economic factors using the available and most recent Demographic and Health Surveys (DHS) data from 2019–2020.

## Methods

### Data source and participants

For the present cross-sectional study, we included all SSA countries that participated in the DHS most recent years (2019–2020) and collected data on MDD among children aged 6–23 months old. There was a total of three countries that had conducted DHS surveys in the years since 2019 and had asked mothers about the types of food their child had consumed during the day or night before the interview. These countries included Gambia, Liberia, and Rwanda. The mean response rate across surveys was 97.9% (range, 99–97.7%). This study followed the Strengthening the Reporting of Observation Studies in Epidemiology (STROBE) reporting guideline (27). Each host country collected data in coordination with ICF International, a global consulting technology services company located in Rockville, MD (28). The DHS surveys are nationally representative household surveys supported by the US Agency for International Development (USAID) for over 30 years. The DHS surveys data included over 300 surveys conducted in more than 90 World Bank-defined LMICs worldwide.

The surveys used multistage cluster sampling and a stratified sampling design to collect detailed information such as sociodemographic characteristics, health behaviors, child's health and nutrition indicators, HIV and AIDS, and reproductive health (29, 30). The first stage involves dividing the country into geographic regions. Then within these regions, populations are stratified either by urban or rural areas. The primary sampling units (PSUs) were selected with a probability proportional to the size within each stratum. All households within the cluster were listed in the second stage of sampling, and approximately 25 households were randomly selected for an interview using equal probability systematic sampling.

The children's records or kid's records (KR) DHS datasets were used for the present study. According to the DHS guideline for assessing MDD among children (https://dhsprogram.com/data/Guide-to-DHS-Statistics/Minimum_Dietary_Diversity.htm), the present weighted analysis was limited to the last-born children aged 6–23 months who were living with their mothers and fed with an MDD during the day or night preceding the survey (*n* = 5,832).

### Study variables

#### Outcome variables

The MDD is a population-level indicator designed by the WHO to assess diet diversity among children 6–23 months old. This indicator is one of the eight IYCF indicators developed by the WHO to provide simple, valid, and reliable metrics for determining IYCF practices (31). MDD data are collected from a questionnaire administrated to the child's mothers or caregivers as part of the IYCF module. Based on June 2017 expert consultation, the WHO updated the version of MDD-7 (7 food groups) to MDD-8 to reflect the inclusion of breast milk as an 8^th^ food group. Therefore, the criterion for meeting MDD changed from 4 of 7 groups to 5 of 8 groups (32). The outcome of interest for the present study was the proportion of children's diets meeting MDD during the previous day. According to the most recent WHO (13) and DHS (33), we defined meeting MDD among children aged 6–23 months as at least 5 out of 8 food groups fed during the day or night preceding the survey. The components of the 8 food groups included: (1) breastmilk, (2) grains, roots, and tubers, (3) legumes and nuts, (4) dairy products (infant formula, milk, yogurt, cheese), (5) flesh foods (meat, fish, poultry, and liver/organ meats), (6) eggs, (7) vitamin A-rich fruits and vegetables, (8) other fruits and vegetables. The response options for each food group were 1 for “consumed” and 0 for not “consumed.” A cumulative score was calculated by combining the scores of all the food groups. A binary outcome variable for meeting an MDD was created by assigning “1” for children who consumed ≥ 5 out of 8 food groups and “0” for those who consumed <5 food groups (18).

#### Explanatory variables

We selected country of residence, and child and maternal factors as potential determinants because they have been shown to be correlated with MDD (17, 18, 34). The child's factors included the child's age and sex. The maternal factors included age, antenatal care visits (ANC), household wealth index status, educational status, marital status, place of residence, employment status, household owning a radio, household owning a television, and if visited a healthcare facility in the last 12 months. Both child and maternal factors were collected through self-report questionnaires. Wealth index quintiles were determined using a principal component analysis approach of household assets (household ownership of several items such as television, car, radio, and other wealth-related characteristics). Detailed information on determining wealth index quintiles has been described elsewhere (35). The wealth index was recategorized from quintiles into three categories by combining the poorest and poorer into one category (called “lowest”); middle wealth level into the second category (called “middle”); and richer and richest into the third category (called “highest”), as done in previous studies (36–38). We also recategorized maternal age from a continuous scale into three groups for this study (15–29, 30–39, and 40–49 years old).

### Statistical analysis

We performed statistical analyses using SAS statistical software version 9.4 (SAS Institute, Cary, NC, USA) and R version 3.4.3 (R Foundation for Statistical Computing, Vienna, Austria). Consistent with the DHS guideline for analyzing the DHS data and to ensure that the estimates were nationally representative, all analyses were conducted using appropriate survey weights, clustering, and stratification to account for the complex sampling design (39). Univariable analyses were performed using frequency distributions for categorical variables to describe the characteristics of the study participants. The prevalence of meeting MDD was calculated as the number of children who met the MDD divided by the total number of children in that category multiplied by 100%. Multivariable logistic regression models (proc surveylogistic; SAS institute) were used to examine each independent factor's association with meeting MDD. A stratified analysis was also conducted to examine the prevalence of each of the 8 nutrient-rich food groups described above by country. In addition, to better understand between-country differences, we also analyzed each demographic/social factor of MDD stratified by country. A Variance Inflation Factor (VIF) was performed to measure the degree of multicollinearity among independent variables, which did not indicate any substantial multicollinearity from the full adjusted model, with VIF values of 3 or less. Among our selected factors, 8 participants had missing data for ANC visits, 157 participants for access to a radio, and 157 participants for access to a TV. Considering that the proportion of missing data was very low (2.6%), a complete case analysis approach was adopted. To test the robustness of our results, we also conducted a sensitivity analysis using a multivariable binomial regression in which the outcome variable was the number of food groups (numerator) divided by eight (denominator). Descriptive statistics are presented as the weighted prevalence of meeting MDD, and the multivariable logistic regression results are presented as adjusted odds ratios (OR) with 95% confidence intervals (CIs). Statistical tests were reported as significant at *p*-values <0.05, and all *p*-values were 2-sided.

### Ethical considerations

Each country's procedures and questionnaires for standard DHS surveys were reviewed and approved by the ICF International Institutional Review Board (IRB) and the IRBs of each host country. Before the survey, written or oral informed consent was obtained from each participant or proxy. Survey participants were not coerced into participation (40), and all data are completely de-identified with no names or household addresses in the data files. Thus, no further IRB approval was needed by the authors' institutions of the present manuscript. Details on the ethical matters are described in the DHS methodology, protecting the Privacy of DHS Survey Respondents (41).

## Results

### Sociodemographic characteristics of the participants

A total of 5,832 children aged 6–23 months from three SSA who live with their mothers were included in this analysis (Table 1). The mean age (SE) of the children was 14.2 (0.01) months. The majority of the children were between 6–11 months and were males (51.0%). More than one-half of these children's mothers were younger (15–29 years old) (54.5%), had four or more antenatal care visits than <4 (69.7%), and were mostly from the lowest household wealth index status (44.1%) than the middle and highest. In addition, more than one-half of mothers had access to a radio (51.0%), visited a healthcare facility in the last 12 months (82.5%), lived in rural areas (57.4%), and were employed (63.6%) (Table 2).

**Table 1:** Background characteristics of the weighted survey participants, the prevalence of meeting minimum dietary diversity by country and survey year (*N* = 5,832).

			**All participants**	**Minimum dietary diversity**
**Countries**	**Survey year**	**Response rate (%)**	*N*^a^ **(%**^b^**)**	*N*^c^ **(%)**
**Overall**			5,832	1,356 (23.3)
Liberia	2019–2020	99	1,360 (23.3)	117 (8.6)
Gambia	2019–2020	97	2,109 (36.2)	425 (20.2)
Rwanda	2019–2020	97.7	2,363 (40.5)	814 (34.4)

N^a^, Weighted sample size of the combined dataset that is represented by that survey for each country.

%^b^, The % of the combined dataset represented by that survey.

N^c^, Prevalence of minimum dietary diversity.

**Table 2:** Background characteristics of the weighted survey participants, the prevalence of meeting minimum dietary diversity, and the multivariable-adjusted odds ratio (*N* = 5,832).

	**All participants**	**Minimum dietary diversity**	**Multivariable-adjusted analysis**	
**Characteristics**	*N*^a^ **(%**^b^**)**	*N*^c^ **(%)**	**(OR) (95% CI)**	***p*** **-value**
**Child characteristics**				
**Age of child**				
6–11 months	2,070 (35.5)	342 (16.5)	ref.	
12–17 months	2,020 (34.6)	539 (26.7)	1.96 (1.61, 2.39)	<0.001
18–23 months	1,742 (30.0)	474 (27.2)	1.92 (1.58, 2.33)	<0.001
**Sex of child**				
Male	2,958 (50.7)	697 (23.6)	ref.	
Female	2,874 (49.3)	658 (22.9)	0.97 (0.83, 1.15)	0.75
**Maternal factors**				
**Age groups**				
15–29	3,176 (54.5)	675 (21.3)	ref.	
30–39	2,173 (37.3)	551 (25.4)	1.05 (0.88, 1.25)	0.57
40–49	483 (8.3)	129 (26.7)	1.16 (0.87, 1.55)	0.32
**ANC visits**				
<4	1,768 (30.4)	491 (27.8)	ref.	
≥4	4,059 (69.7)	864 (21.3)	0.97 (0.81, 1.17)	0.78
**Wealth index status**				
Lowest	2,572 (44.1)	412 (16.0)	ref.	
Middle	1,177 (20.2)	281 (23.9)	1.45 (1.16, 1.81)	0.001
Highest	2,083 (35.7)	662 (31.8)	1.86 (1.45, 2.39)	<0.001
**Place of residence**				
Urban	2,486 (42.6)	576 (23.2)	ref.	
Rural	3,346 (57.4)	779 (23.3)	0.90 (0.71, 1.14)	0.37
**Maternal education**				
No education	1,617 (27.7)	230 (14.2)	ref.	
Primary	2,294 (39.3)	573 (25.0)	1.14 (0.91, 1.43)	0.24
Secondary/Higher	1,921 (32.9)	552 (28.7)	1.69 (1.35, 2.12)	<0.001
**Marital status**				
Never married	700 (12.0)	131 (18.7)	ref.	
Married/Living with partner	4,848 (83.1)	1,161 (23.9)	1.09 (0.81, 1.47)	0.55
Widowed/Divorced/Separated	284 (4.9)	63 (22.2)	1.13 (0.71, 1.78)	0.61
**Maternal employment**				
No	2,125 (36.5)	423 (19.9)	ref.	
Yes	3,707 (63.6)	932 (25.1)	1.20 (1.01, 1.44)	0.04
**Household has radio**				
No	2,790 (49.1)	560 (20.1)	ref.	
Yes	2,887 (50.9)	765 (26.5)	1.30 (1.09, 1.56)	0.004
**Household has television**				
No	3,887 (68.5)	851 (21.9)	ref.	
Yes	1,791 (31.5)	474 (26.5)	1.00 (0.78, 1.29)	0.99
**Visited healthcare facility last 12 months**			
No	1,020 (17.5)	169 (16.6)	ref.	
Yes	4,812 (82.5)	1,186 (24.6)	1.57 (1.27, 1.92)	<0.001

ANC, Antenatal care.

N^a^, Weighted sample size of the combined dataset.

%^b^, The % of the combined dataset.

N^c^, Prevalence of meeting minimum dietary diversity.

Ref, reference.

**Model** fully adjusted for country of residence, age of the child (categorical), sex of child (male/female), age of mother (categorical), antenatal care visits (< 4/≥ 4), education status (categorical), marital status (categorical), wealth index status (categorical), place of residence (urban/rural), employment status (yes/no), household having a radio (yes/no), household having a television (yes/no), visited health care facility in the last 12 months (yes/no).

### Prevalence of meeting MDD in these SSA countries

Overall, the weighted prevalence of children who met the MDD was 23.2% (95% CI: 21.7%-24.8%), ranging from 8.6% in Liberia to 34.4% in Rwanda (Table 1). The prevalence of meeting MDD among children fed during the day or night preceding the survey was higher among older children aged 12–17 months (26.7%) and 18–23 months (27.2%) compared to aged 6–11 months (16.5%) and males (23.6%) compared to female (22.9%). In addition, maternal factors such as older (40–49 years old) age (26.7%), higher wealth status (31.8%), secondary/higher education (28.7%), employment, access to a radio, and visited healthcare facility in the past 12 months had the highest prevalence of meeting MDD (Table 2).

Country-stratified analysis (Supplementary Table 1) indicated that the prevalence of meeting MDD also varied widely between countries in relation to different maternal factors such as ANC visits, household wealth status, education level, marital status, employment status, access to a radio, and visited healthcare facility in the last 12 months. For all countries, children whose mothers had four or more ANC visits, had the highest household wealth status, were married/living with a partner, were currently employed, had access to a radio, and visited a healthcare facility in the last 12 months had the highest prevalence of meeting MDD consistently. Liberia and Gambia had the highest prevalence of meeting MDD among mothers with secondary/higher education regarding the educational level. For Rwanda, mothers with primary education had the highest prevalence of fulfilling the MDD. For all countries, children aged 12–17 months consistently had the highest prevalence of meeting MDD.

**Figure 1:**
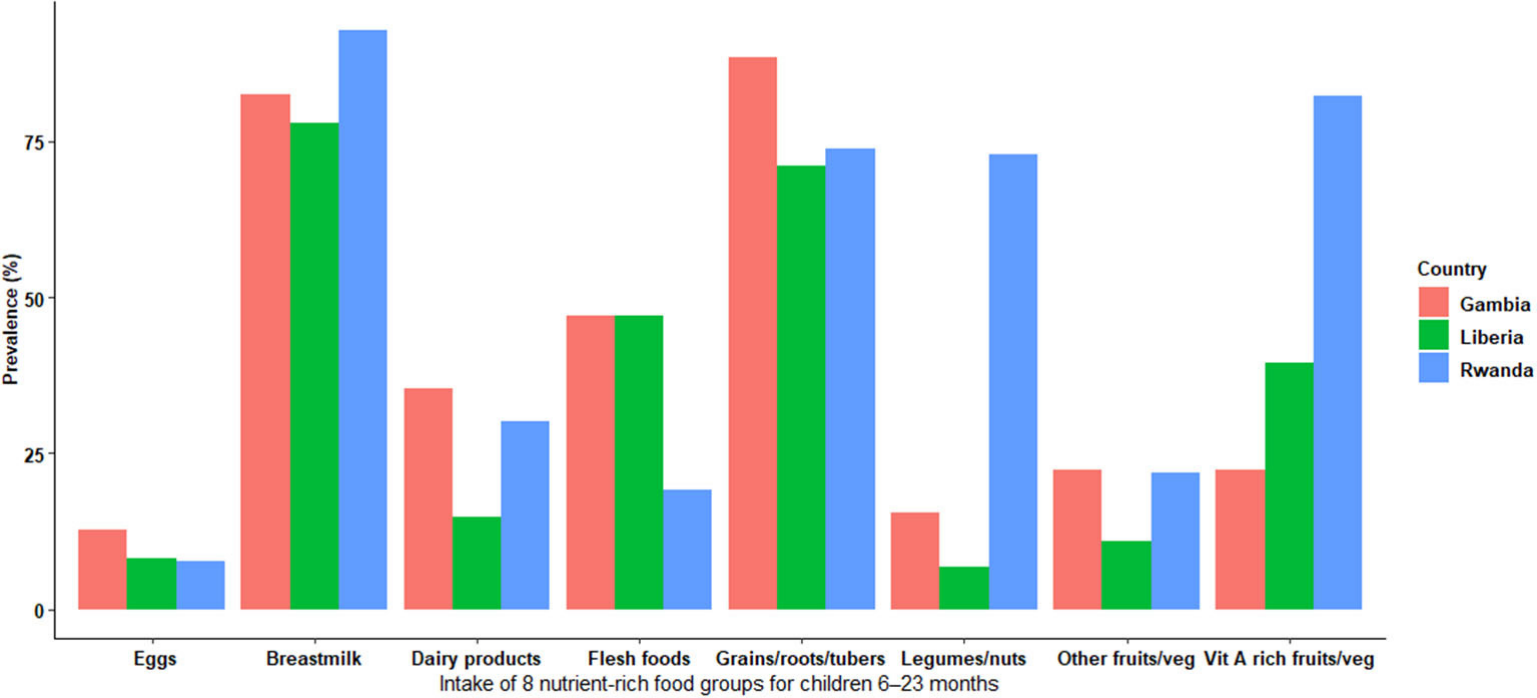
Weighted prevalence of intake of 8 nutrient-rich food groups for children 6–23 months.

Figure 1 shows the prevalence of each of the 8 nutrient-rich food groups included in the MDD stratified by country. There was disparity regarding the 8 nutrients-rich food groups across countries. Rwanda had the highest prevalence of breastmilk, legumes/nuts, and vitamin A-rich fruits/vegetable consumption. Interestingly, the prevalence of receiving protein sources (eggs), dairy products, and other fruits/vegetables was lower in all countries than breastmilk, legumes/nuts, flesh foods, grain/roots/tubes, and vitamin A-rich fruits/vegetables that contribute to low MDD. In addition, eggs were the least food group consumed in all countries. Breastmilk consumption and grains/roots/tubes were consistently higher in all countries than in other food groups (Figure 1).

### Factors associated with meeting MDD

The babies aged 12–17 months were almost 2 times more likely to meet MDD (OR = 1.96; 95% CI: 1.61, 2.39, *p* < 0.001) and aged 18–23 months (OR = 1.92; 95% CI: 1.58, 2.33, *p* < 0.001) when compared to those aged 6–11 months (Table 2). The respondents from the households whose wealth index were middle (OR = 1.45; 95% CI: 1.16, 1.81, *p* = 0.001) and highest (OR = 1.86; 95% CI: 1.45, 2.39, *p* < 0.001) had greater odds to meet MDD than those from the lowest (Table 2). The babies whose mothers had secondary/higher were almost 2 times more likely to meet MDD (OR = 1.69; 95% CI: 1.35, 2.12, *p* < 0.001) compared to those with no formal education (Table 2). Furthermore, the babies whose mothers had employment had higher likelihood to achieve MDD (OR = 1.20; 95% CI: 1.01, 1.44, *p* = 0.04) than those from mothers who had no employment (Table 2). The babies whose mothers had a radio in the households had higher odds to meet MDD (OR = 1.30; 95% CI: 1.09, 1.56, *p* = 0.004) than those who did not have a radio (Table 2). Lastly, children whose mothers visited a healthcare facility in the last 12 months were almost 2 times more likely to meet MDD (OR = 1.57; 95% CI: 1.27, 1.92, *p* < 0.001) (Table 2). Interestingly, we did not observe a significant association between the child's sex and the odds of fulfilling the MDD. These results remained consistent in the sensitivity analysis using multivariable binomial regression.

## Discussion

The purpose of this study was to conduct a population-based cross-sectional study to determine the prevalence of meeting MDD in three combined SSA countries and the associated socio-demographic-economic factors. Our pooled results showed that the mean of the weighted prevalence of children 6–23 months who met the MDD was low (23%) in these three SSA countries and exhibited substantial between-country variation. The low prevalence of meeting MDD among children in these low-resource countries is concerning and has the potential to increase the risk of mortality and chronic diseases in the future. Disparities due to wealth are significant, and children's diets are more likely to meet MDD in wealthier households. More importantly, children whose mothers had more education, were employed, had access to a radio, and visited healthcare facilities in the last 12 months were more likely to meet the MDD. The child's age was also significantly associated with meeting MDD. We found a similar non-significant trend for access to a TV, which could be due to a smaller proportion of households owning a TV. Conversely, we did not observe any significant associations between a child's sex and the odds of meeting MDD. This study showed disparity regarding the 8 nutrients-rich food groups across these three SSA countries. Protein sources such as eggs were the least food group consumed in all countries.

The observed low prevalence of meeting MDD in this study is consistent with a previous study conducted in Ethiopia (17). Lack of food availability or affordability may play a significant role in the low prevalence of meeting MDD observed in this study. Our finding agrees with previous studies that also indicated a significant positive association between a child's age and meeting MDD (18, 42, 43). A potential explanation for this association could be due to older children's willingness to accept diverse foods with different tastes and textures and their familiarity with foods than younger children (18, 44). The positive association between wealthier households and meeting MDD observed in this study is consistent with the findings from previous studies conducted in Ethiopia (17) and Indonesia (18). Consistent with earlier studies from other LMICs (18–20, 42), we found that mothers with higher educational levels were more likely to feed their children with more diversified foods. Socioeconomic inequalities represent a major threat to optimal feeding practices (45). It postulated that poorer households' factors and lack of maternal education regarding an adequate diet for young children could drive these disparities in complementary feeding practices. Therefore, closing the gap in dietary inequalities between countries is critical to preventing long-term socioeconomic and health inequalities. Highly educated mothers might have access to more resources that promote the benefits of a diversified diet and a better understanding of nutritional health education messages (18). This study also found that respondents who had exposure to mass media (radio) had greater odds of achieving MDD. The media such as national radio stations are usually considered to be a reliable source of health and nutrition-related information in low-resource countries, thus its messages are more likely to be embraced (20, 46). This is a similar finding to a study conducted in Ethiopia (17). Additionally, our finding of a significant association between maternal employment and meeting MDD is consistent with previous studies (20–22). Lastly, our observed positive association between mothers who visited a healthcare facility in the last 12 months and the odds of meeting MDD is also consistent with a previous study conducted in Nigeria (21).

### Public health recommendations

The Sustainable Development Goals, part of the call for action toward appropriate diets for children, aim to address goals 2 (zero hunger) and 3 (good health and well-being) (47). Implementing policies and programs to reduce wealth-related inequalities is essential for optimal child nutrition. Recent estimates suggest that more than 11 million cases of stunting could have been averted if the proportion of children's diets meeting MDD was 90% (48). However, in the present study from these three SSA countries, the rates of meeting MDD did not reach the threshold of 50%. Previous studies have observed the critical role of dietary diversity in impacting the relationship with child anthropometry (49, 50).

MDD is a simple yet valid and reliable population-level indicator of IYCF practices and is critical for assessing national and subnational comparisons, and is relevant for identifying populations at risk and targeting interventions. Our current analysis suggests that policies that will effectively increase the prevalence of meeting MDD should target poor households with appropriate materials or financial assistance and mothers with lower literacy. In a recent pooled analysis of 80 low- and middle-income countries, dietary diversity was higher when absolute household income exceeded ~US$20,000 (51). Additionally, public health interventions working with other sectors such as education and radio stations to promote health education about the benefits of diverse diets (18), especially among teenage girls, are critically needed. Targeting teenage girls with nutrition-related interventions before becoming pregnant may significantly increase the prevalence of meeting MDD. More importantly, providing financial assistance to poorer households or the availability of food pantries may also play an essential role in ensuring each child consumes adequately diverse foods to meet their nutritional requirements (34).

### Study strengths and limitations

The strength of this study is the analysis of a nationally representative sample of children aged 6–23 months from three SSA countries using 2019–2020 DHS data. To the best of our knowledge, this is one of the few comprehensive studies to investigate the prevalence and determinants of meeting MDD using the most recent DHS data across multiple SSA countries with high response rates. In addition, we used the most updated MDD indicator with eight food groups, which is a valid and reliable metric for assessing IYCF feeding practices at the population level developed by the WHO (9).

Notwithstanding, the present study has a few limitations that are worth mentioning. First, the cross-sectional nature of the survey does not allow for determining causality. Secondly, almost all low-income countries are found in SSA. Most recent MDD data was limited to only three of the 48 countries in SSA, and thus, our findings may lack external validity for other SSA low-income countries. Moreover, MDD was based on maternal recall, which may be subject to recall bias and social desirability (18, 25). Additionally, this study did not adjust for total energy due to a lack of data on calorie consumption from the DHS database. Lastly, a single 24-diet recall is not considered to be representative of habitual diet at an individual level. Because it doesn't account for day-to-day variation.

## Conclusions

In this study using three SSA countries, few children were fed a diet that met MDD on the day of recall. Interestingly, the prevalence of eggs, dairy products, and other fruits/vegetables being consumed remained very low in all countries. Maternal education, household wealth status, employment status, access to a radio, visited healthcare facilities in the last 12 months, and age of the child was the significant determinant of meeting the WHO recommended feeding practice indicator of MDD among the youngest children in these three SSA countries. We did not observe a significant association between the child's sex and the odds of fulfilling the MDD. The findings highlighted the need to target mothers, especially those with low education and lower household wealth status, through health education about the importance of adequately diversified foods and financial assistance to ensure optimal child growth in these low-resource countries and prevent undernutrition.

## Data availability statement

Publicly available datasets were analyzed in this study. This data can be found at: https://dhsprogram.com/data/.

## Author contributions

Designed research (project conception, development of overall research plan, and study oversight) and analyzed data, or performed statistical analysis: DB. Wrote the first draft of the manuscript: DB and PS. All authors reviewed and commented on subsequent drafts of the manuscript and have read and approved the final version of the manuscript.

## Conflict of interest

The authors declare that the research was conducted in the absence of any commercial or financial relationships that could be construed as a potential conflict of interest.

## Publisher's note

All claims expressed in this article are solely those of the authors and do not necessarily represent those of their affiliated organizations, or those of the publisher, the editors and the reviewers. Any product that may be evaluated in this article, or claim that may be made by its manufacturer, is not guaranteed or endorsed by the publisher.

## References

[1] Lim SS Vos T Flaxman AD Danaei G Shibuya K Adair-Rohani H. A comparative risk assessment of burden of disease and injury attributable to 67 risk factors and risk factor clusters in 21 regions, 1990–2010: a systematic analysis for the global burden of disease study 2010. Lancet. (2012) 380:2224–60. 10.1016/S0140-6736(12)61766-8 23245609 PMC4156511

[2] Akombi BJ Agho KE Merom D Renzaho AM Hall JJ. Child malnutrition in sub-Saharan Africa: a meta-analysis of Demographic and Health Surveys (2006–2016). PLoS ONE. (2017) 12:e0177338. 10.1371/journal.pone.0177338 28494007 PMC5426674

[3] UNICEF Data: Monitoring the situation of children women. Malnutrition. Available online at https://data.unicef.org/topic/nutrition/malnutrition/ (accessed July 25, 2022).

[4] Benson T Shekar M. Trends and Issues in Child Undernutrition. In: Jamison DT Feachem RG Makgoba MW Bos ER Baingana FK Hofman KJ., editors. Disease and Mortality in Sub-Saharan Africa. 2nd ed. Washington, DC: The International Bank for Reconstruction and Development / The World Bank (2006). 21290662

[5] Black RE Victora CG Walker SP Bhutta ZA Christian P de Onis M. Maternal and child undernutrition and overweight in low-income and middle-income countries. Lancet. (2013) 382:427–51. 10.1016/S0140-6736(13)60937-X 23746772

[6] UNICEF. Malnutrition Rates Remain Alarming: Stunting is Declining Too Slowly While Wasting Still Impacts the Lives of Far Too Many Young Children. (2018). Available online at: http://data.unicef.org/topic/nutrition/malnutrition/#

[7] World Health Organization. The UNICEF/WHO/WB Joint Child Malnutrition Estimates (JME) Group Released New Data for 2021. Available online at: https://www.who.int/news/item/06-05-2021-the-unicef-who-wb-joint-child-malnutrition-estimates-group-released-new-data-for-2021 (accessed December 12, 2021).

[8] World Health Organization. Global Health Observatory (GHO) Data. (2016). Available online at: http://www.who.int/gho/child_health/mortality/mortality_under_five/en/

[9] Working Group on Infant and Young Child Feeding Indicators. Developing and Validating Simple Indicators of Dietary Quality and Energy Intake of Infants and Young Children in Developing Countries: Summary of Findings From Analysis of 10 Data Sets. Washington, DC: Food and Nutrition Technical Assistance Project (FANTA) (2006).

[10] Moursi MM Arimond M Dewey KG Trèche S Ruel MT Delpeuch F. Dietary diversity is a good predictor of the micronutrient density of the diet of 6–23-month-old children in Madagascar. J Nutr. (2008) 138:2448–53. 10.3945/jn.108.093971 19022971

[11] Solomon D Aderaw Z Tegegne TK. Minimum dietary diversity and associated factors among children aged 6-23 months in Addis Ababa, Ethiopia. Int J Equity Health. (2017) 16:181. 10.1186/s12939-017-0680-1 29025434 PMC5639776

[12] World Health Organization. Indicators For Assessing Infant and Young Child Feeding Practices: Part 1: Definitions: Conclusions of a Consensus Meeting Held 6–8 November 2007 in Washington DC, USA. (2008). Available online at: https://apps.who.int/iris/bitstream/handle/10665/43895/9789241596664_eng.pdf?sequence (accessed December 12, 2021).

[13] World Health Organization (WHO). Global Nutrition Monitoring Framework: Operational Guidance for Tracking Progress in Meeting Targets for 2025. (2017). Available online at: https://www.who.int/publications/i/item/9789241513609 (accessed December 12, 2021).

[14] Temesgen H Yeneabat T Teshome M. Dietary diversity and associated factors among children aged 6–23 months in Sinan Woreda, Northwest Ethiopia: a cross-sectional study. BMC Nutrition. (2018) 4:5. 10.1186/s40795-018-0214-2 32153869 PMC7050891

[15] Kennedy GL Pedro MR Seghieri C Nantel G Brouwer I. Dietary diversity score is a useful indicator of micronutrient intake in non-breast-feeding Filipino children. J Nutr. (2007) 137:472–7. 10.1093/jn/137.2.472 17237329

[16] The Food and Nutrition Technical Assistance III Project (FANTA) [2012-2018]. Why is dietary diversity important? Available online at: https://www.fantaproject.org/node/1199 (accessed December 13, 2021).

[17] Eshete T Kumera G Bazezew Y Mihretie A Marie T. Determinants of inadequate minimum dietary diversity among children aged 6–23 months in Ethiopia: secondary data analysis from Ethiopian demographic and health survey 2016. Agric Food Secur. (2018) 7:66. 10.1186/s40066-018-0219-8

[18] Paramashanti B Huda T Alam A Dibley M. Trends and determinants of minimum dietary diversity among children aged 6–23 months: A pooled analysis of Indonesia Demographic and Health Surveys from 2007 to 2017. Public Health Nutr. (2022) 25:1956–967. 10.1017/S1368980021004559 34743776 PMC9991623

[19] Aemro M Mesele M Birhanu Z Atenafu A. Dietary diversity and meal frequency practices among infant and young children aged 6-23 months in Ethiopia: a secondary analysis of Ethiopian demographic and health survey 2011. J Nutr Metab. (2013) 2013:782931. 10.1155/2013/782931 24455218 PMC3878383

[20] Nkoka O Mhone TG Ntenda PAM. Factors associated with complementary feeding practices among children aged 6-23 mo in Malawi: an analysis of the demographic and health survey 2015–2016. Int Health. (2018) 10:466–79. 10.1093/inthealth/ihy047 30052967

[21] Ogbo FA Page A Idoko J Claudio F Agho KE. Trends in complementary feeding indicators in Nigeria, 2003–2013. BMJ Open. (2015) 5:e008467. 10.1136/bmjopen-2015-008467 26443657 PMC4606380

[22] Issaka AI Agho KE Page AN Burns PL Stevens GJ Dibley MJ. Determinants of suboptimal complementary feeding practices among children aged 6–23 months in seven francophone West African countries. Matern Child Nutr. (2015) 11:31–52. 10.1111/mcn.12193 26364790 PMC6860307

[23] Molla M Ejigu T Nega G. Complementary feeding practice and associated factors among mothers having children 6–23 months of age, Lasta District, Amhara Region, Northeast Ethiopia. Adv Public Health. (2017) 2017:4567829. 10.1155/2017/4567829

[24] Egbuonye NC Ishdorj A McKyer ELJ Mkuu R. Examining the dietary diversity of children in Niger. Nutrients. (2021) 13:2961. 10.3390/nu13092961 34578839 PMC8467481

[25] Tegegne M Sileshi S Benti T Teshome M Woldie H. Factors associated with minimal meal frequency and dietary diversity practices among infants and young children in the predominantly agrarian society of Bale zone, Southeast Ethiopia: a community based cross sectional study. Arch Public Health. (2017) 75:53. 10.1186/s13690-017-0216-6 29158896 PMC5682638

[26] Ochieng J Afari-Sefa V Lukumay PJ Dubois T. Determinants of dietary diversity and the potential role of men in improving household nutrition in Tanzania. PLoS ONE. (2017) 12:e0189022. 10.1371/journal.pone.0189022 29232413 PMC5726653

[27] von Elm E Altman DG Egger M Pocock SJ Gøtzsche PC Vandenbroucke JP. Strengthening the Reporting of Observational Studies in Epidemiology (STROBE) statement: guidelines for reporting observational studies. BMJ. (2007) 335:806–8. 10.1136/bmj.39335.541782.AD 17947786 PMC2034723

[28] Kent EE Forsythe LP Yabroff KR Weaver KE de Moor JS Rodriguez JL. Are Survivors who report cancer-related financial problems more likely to forgo or delay medical care? Cancer-Am Cancer Soc. (2013) 119:3710–7. 10.1002/cncr.28262 23907958 PMC4552354

[29] Rutstein S Rojas G. Guide to DHS statistics. Demographic and Health Surveys. Calverton, MD: ORCMacro (2003).

[30] Macro International Inc. Measure DHS: Demographic and Healthsurveys. Available online at: http://www.measuredhs.com/countries/browse_country.cfm?selected=2

[31] World Health Education. Indicators for Assessing Infant and Young Child Feeding Practices. Available online at: http://apps.who.int/iris/bitstream/handle/10665/43895/9789241596664_eng.pdf (accessed July 25, 2022).

[32] INDDEX Project. Data4Diets: Building Blocks for Diet-related Food Security Analysis. Boston, MA: Tufts University (2018).

[33] Guide to DHS Statistics DHS-7. Minimum Dietary Diversity, Minimum Meal Frequency and Minimum Acceptable Diet. Available online at: https://dhsprogram.com/data/Guide-to-DHS-Statistics/Minimum_Dietary_Diversity_Minimum_Meal_Frequency_and_Minimum_Acceptable_Diet.htm (accessed July 25, 2022).

[34] Sekartaji R Suza DE Fauziningtyas R Almutairi WM Susanti IA Astutik E. Dietary diversity and associated factors among children aged 6–23 months in Indonesia. J Pediatr Nurs. (2021) 56:30–4. 10.1016/j.pedn.2020.10.006 33181370

[35] Rutstein SO. Steps to Constructing the New DHS Wealth Index. Rockville, MD: ICF International (2015).

[36] Lunani LL Abaasa A Omosa-Manyonyi G. Prevalence and factors associated with contraceptive use among Kenyan women aged 15–49 years. AIDS Behav. (2018) 22:125–30. 10.1007/s10461-018-2203-5 29943124 PMC6132050

[37] Titilayo A Palamuleni ME Olaoye-Oyesola JO Owoeye OM. Religious perceptions and attitudes of men towards discontinuation of female genital cutting in Nigeria: evidence from the 2013 Nigeria demographic and health survey. Afr J Reprod Health. (2018) 22:20–8. 10.29063/ajrh2018/v22i1.2 29777639

[38] Ba DM Ssentongo P Kjerulff KH Na M Liu G Gao X. Adherence to iron supplementation in 22 Sub-Saharan African countries and associated factors among pregnant women: a large population-based study. Curr Dev Nutr. (2019) 3:nzz120. 10.1093/cdn/nzz120 31777771 PMC6867960

[39] Macro International Inc. Measure DHS: Demographic and Healthsurveys. Using Datasets for Analysis. Available online at: https://www.dhsprogram.com/data/Using-Datasets-for-Analysis.cfm (accessed June 13, 2020).

[40] Mishra V Vaessen M Boerma JT Arnold F Way A Barrere B. Testing in national population-based surveys: experience from the Demographic and Health Surveys. Bull World Health Organ. (2006) 84:537–45. 10.2471/BLT.05.029520 16878227 PMC2627390

[41] Macro International Inc. Measure DHS: Demographic and Healthsurveys. Available online at: https://dhsprogram.com/What-We-Do/Protecting-the-Privacy-of-DHS-Survey-Respondents.cfm (accessed June 13, 2020).

[42] Harvey CM Newell ML Padmadas SS. Socio-economic differentials in minimum dietary diversity among young children in South-East Asia: evidence from Demographic and Health Surveys. Public Health Nutr. (2018) 21:3048–57. 10.1017/S1368980018002173 30178732 PMC6190069

[43] Beyene M Worku AG Wassie MM. Dietary diversity, meal frequency and associated factors among infant and young children in Northwest Ethiopia: a cross- sectional study. BMC Public Health. (2015) 15:1007. 10.1186/s12889-015-2333-x 26433689 PMC4592571

[44] Mura Paroche M Caton SJ Vereijken C Weenen H Houston-Price C. How infants and young children learn about food: a systematic review. Front Psychol. (2017) 8:1046. 10.3389/fpsyg.2017.01046 28790935 PMC5524770

[45] Gibbs BG Forste R. Socioeconomic status, infant feeding practices and early childhood obesity. Pediatr Obes. (2014) 9:135–46. 10.1111/j.2047-6310.2013.00155.x 23554385

[46] Dangura D Gebremedhin S. Dietary diversity and associated factors among children 6-23 months of age in Gorche district, Southern Ethiopia: cross-sectional study. BMC Pediatr. (2017) 17:6. 10.1186/s12887-016-0764-x 28068965 PMC5223415

[47] Willis K. The sustainable development goals. In:The Routledge Handbook of Latin American Development. London: Taylor & Francis Group (2018). p. 121–31.

[48] Baye K Kennedy G. Estimates of dietary quality in infants and young children (6-23 mo): evidence from Demographic and Health Surveys of 49 low- and middle-income countries. Nutrition. (2020) 78:110875. 10.1016/j.nut.2020.110875 32653760

[49] Bosha T Lambert C Riedel S Melesse A Biesalski HK. Dietary diversity and anthropometric status of mother-child pairs from enset (false banana) staple areas: a panel evidence from Southern Ethiopia. Int J Environ Res Public Health. (2019) 16:2170. 10.3390/ijerph16122170 31248176 PMC6617300

[50] Dinku AM Mekonnen TC Adilu GS. Child dietary diversity and food (in)security as a potential correlate of child anthropometric indices in the context of urban food system in the cases of north-central Ethiopia. J Health Popul Nutr. (2020) 39:11. 10.1186/s41043-020-00219-6 33298197 PMC7771062

[51] Gatica-Domínguez G Neves PAR Barros AJD Victora CG. Complementary feeding practices in 80 low- and middle-income countries: prevalence of and socioeconomic inequalities in dietary diversity, meal frequency, and dietary adequacy. J Nutr. (2021) 151:1956–64. 10.1093/jn/nxab088 33847352 PMC8245881

