# Supplementary material for: Prevalence and determinants of meeting minimum dietary diversity among children aged 6–23 months in three sub-Saharan African Countries: The Demographic and Health Surveys, 2019–2020
Source: Front Public Health. 2022 Aug 23;10:846049. doi: 10.3389/fpubh.2022.846049 (PMC9445207; doi:10.3389/fpubh.2022.846049)
Supplement: Supplementary file 1 [file Table_1.DOCX]

**Supplemental Table 1. Prevalence of meeting MDD stratify by country (N=5,832)**

|  | Liberia  (N=1,360) | Gambia  (N=2,109) | Rwanda  (N=2,363) |
| --- | --- | --- | --- |
| **Characteristic** | **N (%)** | **N (%)** | **N (%)** |
| **Child Factors** |  |  |  |
| **Age of child** |  |  |  |
| 6-11 months | 25 (1.83) | 58 (2.75) | 259 (10.96) |
| 12-17 months | 55 (4.04) | 197 (9.34) | 286 (12.10) |
| 18-23 months | 38 (2.79) | 169 (8.01) | 268 (11.34) |
| **Sex of child** |  |  |  |
| Male | 53 (3.90) | 229 (10.86) | 415 (17.56) |
| Female | 64 (4.71) | 196 (9.29) | 398 (16.84) |
| **Maternal Factors** |  |  |  |
| **Age groups** |  |  |  |
| 15-29 | 73 (5.36) | 234 (11.10) | 368 (15.57) |
| 30-39 | 32 (2.35) | 162 (7.68) | 357 (15.11) |
| 40-49 | 13 (0.95) | 28 (1.32) | 88 (3.72) |
| **ANC visits** |  |  |  |
| < 4 | 13 (0.95) | 89 (4.22) | 389 (16.46) |
| ≥ 4 | 105 (7.72) | 335 (15.88) | 425 (18.00) |
| **Wealth index status** |  |  |  |
| Lowest | 42 (3.09) | 139 (6.59) | 231 (9.78) |
| Middle | 25 (1.84) | 113 (5.36) | 143 (6.05) |
| Highest | 50 (3.68) | 173 (8.20) | 439 (18.58) |
| **Place of residence** |  |  |  |
| Urban | 78 (5.74) | 313 (14.84) | 186 (7.87) |
| Rural | 40 (2.94) | 112 (5.31) | 628 (26.58) |
| **Maternal Education** |  |  |  |
| No education | 25 (1.84) | 155 (7.35) | 49 (2.07) |
| Primary | 33 (2.43) | 96 (4.55) | 445 (18.83) |
| Secondary/Higher | 60 (4.41) | 173 (8.20) | 320 (13.54) |
| **Marital status** |  |  |  |
| Never married | 30 (2.20) | 17 (0.81) | 85 (3.60) |
| Married/Living with partner | 79 (5.81) | 398 (18.87) | 685 (28.99) |
| Widowed/Divorced/Separated | 9 (0.66) | 10 (0.47) | 44 (1.86) |
| **Maternal Employment** |  |  |  |
| No | 46 (3.38) | 155 (7.35) | 22 (9.39) |
| Yes | 72 (5.29) | 270 (12.78) | 591 (25.01) |
| **Household has radio** |  |  |  |
| No | 56 (4.12) | 117 (5.58) | 386 (16.34) |
| Yes | 58 (4.26) | 293 (13.89) | 414 (17.52) |
| **Household has television** |  |  |  |
| No | 86 (6.32) | 137 (6.50) | 628 (26.58) |
| Yes | 28 (2.06) | 274 (12.99) | 171 (7.24) |
| **Visited healthcare facility last 12mths** |  |  |  |
| No | 27 (1.98) | 20 (0.95) | 123 (5.21) |
| Yes | 91 (6.69) | 405 (19.20) | 690 (29.20) |

Note: we only reported the number (%) of meeting MDD for each variable by country stratify. The numbers are not meant to sum up. We used the total number for each country as the denominator when calculating the prevalence of meeting MDD.
